# Experimental Investigation on Thermophysical Properties of Ammonium-Based Protic Ionic Liquids and Their Potential Ability towards CO_2_ Capture

**DOI:** 10.3390/molecules27030851

**Published:** 2022-01-27

**Authors:** Nur Hidayah Zulaikha Othman Zailani, Normawati M. Yunus, Asyraf Hanim Ab Rahim, Mohamad Azmi Bustam

**Affiliations:** 1 Department of Fundamental and Applied Sciences, Centre of Research in Ionic Liquids (CORIL), Institute of Contaminant Management for Oil and Gas, Universiti Teknologi PETRONAS, Seri Iskandar 32610, Malaysia; zulaikha95.nhz@gmail.com (N.H.Z.O.Z.); asyrafhanim92@gmail.com (A.H.A.R.); 2 Department of Chemical Engineering, Centre of Research in Ionic Liquids (CORIL), Institute of Contaminant Management for Oil and Gas, Universiti Teknologi PETRONAS, Seri Iskandar 32610, Malaysia; azmibustam@utp.edu.my

**Keywords:** ammonium-based protic ionic liquids, density, viscosity, refractive index, phase transition, thermal expansion coefficient, standard entropy, lattice potential energy, CO_2_ absorption

## Abstract

Ionic liquids, which are extensively known as low-melting-point salts, have received significant attention as the promising solvent for CO_2_ capture. This work presents the synthesis, thermophysical properties and the CO_2_ absorption of a series of ammonium cations coupled with carboxylate anions producing ammonium-based protic ionic liquids (PILs), namely 2-ethylhexylammonium pentanoate ([EHA][C5]), 2-ethylhexylammonium hexanoate ([EHA][C6]), 2-ethylhexylammonium heptanoate ([EHA][C7]), bis-(2-ethylhexyl)ammonium pentanoate ([BEHA][C5]), bis-(2-ethylhexyl)ammonium hexanoate ([BEHA][C6]) and bis-(2-ethylhexyl)ammonium heptanoate ([BEHA][C7]). The chemical structures of the PILs were confirmed by using Nuclear Magnetic Resonance (NMR) spectroscopy while the density (*ρ*) and the dynamic viscosity (*η*) of the PILs were determined and analyzed in a range from 293.15K up to 363.15K. The refractive index (*n*_D_) was also measured at T = (293.15 to 333.15) K. Thermal analyses conducted via a thermogravimetric analyzer (TGA) and differential scanning calorimeter (DSC) indicated that all PILs have the thermal decomposition temperature, T_d_ of greater than 416K and the presence of glass transition, T_g_ was detected in each PIL. The CO_2_ absorption of the PILs was studied up to 29 bar at 298.15 K and the experimental results showed that [BEHA][C7] had the highest CO_2_ absorption with 0.78 mol at 29 bar. The CO_2_ absorption values increase in the order of [C5] < [C6] < [C7] anion regardless of the nature of the cation.

## 1. Introduction

Natural gas is a naturally occurring hydrocarbon that consists of methane gas primarily followed by other mixtures of higher alkanes such as ethane, propane and butane. Generally, natural gas is widely used as a fuel and a raw material in the petrochemical industry [1,2]. Despite its mixture of combustible hydrocarbons content, trace quantities of argon (Ar), hydrogen (H), helium (He), nitrogen (N_2_) as well as carbon dioxide (CO_2_) and hydrogen sulfide (H_2_S) are also present in natural gas [3]. Sour gas, such as CO_2_, is undesirable due to its acidic property that causes corrosion in the gas pipeline [4]. Apart from that, the existence of CO_2_ also reduces the fuel value of natural gas due to its non-combustible nature. Therefore, CO_2_ removal in the refining process is crucial to improving the value of natural gas and the utilization of amine-based solvents, namely monoethanolamine (MEA), which had been widely practiced on industrial scales to capture CO_2_ in natural gas. This chemical absorption of CO_2_ by MEA is considered to be the most reliable and efficient technology for capturing CO_2_ [5,6,7,8]. Gómez-Díaz and his team had compared the ability of their blended amine solvent, which is diamine (N,N-dimethylethylenediamine [DMEDA]) with MEA, towards CO_2_ capture in which changes in the amine ratios did not lead to important changes in the absorption curve [6]. Despite the outstanding performance of amine-based solvents, it is also known to have a high vapor pressure and high energy input for regeneration. Therefore, studies related to the utilization of solid adsorbents such as zeolites, activated carbon, amine-functionalized adsorbents and metal organic frameworks (MOFs) had been conducted for CO_2_ adsorption due to their uniqueness as they can be personalized to capture CO_2_ from either post- or pre-combustion gas streams, depending upon several factors [9]. Current examples of adsorbents for CO_2_ adsorption are zeolites, activated carbon, amine-functionalized adsorbents and metal organic frameworks (MOFs) [10,11,12]. Nonetheless, further analysis using adsorbents showed poor adsorption characteristics at low CO_2_ partial pressures [13,14]. Furthermore, membrane separation processes are also used commercially for CO_2_ removal from natural gas. However, a single-stage membrane system is not capable of capturing CO_2_ with high efficiency [15,16,17,18,19,20]. Due to the given issue, this had encouraged researchers to find alternative solvents that can capture CO_2_.

Recently, ionic liquids have been recognized as promising solvents for CO_2_ capture from natural gas. The uniqueness of their properties, specifically their non-detectable vapor pressure, high thermal stability, and high affinity for CO_2,_ enables ILs to be used as solvents for CO_2_ capture at elevated temperatures and pressures [13,21,22,23,24,25]. Moreover, the chemical and physical properties of ILs can be altered due to the availability of countless cation and anion combinations. Several studies involving mainly binary systems of imidazolium-based ILs-CO_2_ or imidazolium-based ILs-other gas have revealed the significant solubility of CO_2_ in ionic liquids when compared to other gases. For example, comparison studies of CO_2_ absorption in individual solvents of 30 wt% of 1-(3-aminopropyl)-3-(2-aminoethyl)imidazolium hydroxide [Apaeim][OH], 30 wt% of 1-(3-aminopropyl)-3-(2-aminoethyl)imidazolium alaninate ([Apaeim][ala]) and 30 wt% monoethanolamine (MEA), have shown that both ILs displayed higher CO_2_ absorption capacities than that of MEA solvent by the value of 2.2-fold. This has further proven that ILs are promising solvents for CO_2_ capture [26]. In addition, a different class of ionic liquids namely fluorine-based protic ILs (FPILs) have displayed competitive properties for selective removal of CO_2_ from flue gas and natural gas [27]. Regardless of the promising performance of CO_2_ capture demonstrated by these types of ionic liquids, they are relatively expensive, they require several steps in the synthesis process, and the utilization of volatile organic solvents is inevitable during the purification process. Recently, protic ionic liquids (PILs) have attracted great interest because of their low cost and simple synthesis pathway. Generally, PILs can be conveniently prepared from stoichiometric neutralization between Brönsted acids and bases. Besides this, PILs display similar CO_2_ absorptivity with other classes of ionic liquids [28,29,30,31,32,33]. In addition, Zhu and his team have synthesized a new PIL from superbase 1,8-diazabicyclo [5.4.0]- undec-7-ene (DBU) with imidazole, and they found that the PIL could reversibly capture about 1 mole of CO_2_ per mole ionic liquid [34]. However, prior to utilization of ionic liquids for any applications, their precise and reliable basic thermophysical properties such as density, viscosity, thermal stability and thermal expansion data are vital for the design and scale up of process equipment. For example, density and thermal expansion data are essential for equipment sizing while thermal stability is required to ensure the practicality of the operating temperature range [35]. In addition, data on solvents’ viscosity is important for the designing of industrial processes related to heat and mass transfer as well as dissolution of compounds in solvents [36]. Several research groups have also investigated and provided discussion on the temperature-dependent properties of protic ionic liquids prior to the utilization of ionic liquids in various applications [19,22,31].

Despite promising results of CO_2_ absorption by protic ionic liquids published in the literature [34], our current work is focusing on the utilization of much cheaper starting reagents, namely amine solutions, for the production of new ammonium-based protic ionic liquids. This work serves as a continuation from our previous work on CO_2_ absorption utilizing ammonium-based protic ionic liquids (PILs) [37]. Previously, the CO_2_ absorption of ammonium-based PILs utilizing bis (2-ethylhexyl) ammonium, tributylammonium and ethanolammonium cations coupled with acetate and butyrate anions have been reported. The motivation to further investigate this type of ionic liquid for CO_2_ capture has risen after we discovered that the PILs could be prepared via a simple synthesis procedure and their capability to absorb CO_2_ under experimental conditions. To further study the binary system of PILs–CO_2_, the synthesis of six new ammonium-based PILs, namely 2-ethylhexylammonium pentanoate ([EHA][C5]), 2-ethylhexylammonium hexanoate ([EHA][C6]), 2-ethylhexylammonium heptanoate ([EHA][C7]), bis-(2-ethylhexyl)ammonium pentanoate ([BEHA][C5]), bis-(2-ethylhexyl)ammonium hexanoate ([BEHA][C6]) and bis-(2-ethylhexyl)ammonium heptanoate ([BEHA][C7]) and their performance towards CO_2_ absorption in a pressure range from 1 bar to 29 bar at 298.15K, are reported in this work.

## 2. Results and Discussion

### 2.1. Characterization of Synthesized PILs

All six ammonium-based PILs in this work exist as liquids at room temperature. The ammonium cation, [EHA] and [BEHA] were combined with anions from organic acids, [C5], [C6] and [C7] through acid-base neutralization reactions. The NMR results and the water contents for all six ammonium-based PILs, [EHA][C5], [EHA][C6], [EHA][C7], [BEHA][C5], [BEHA][C6] and [BEHA][C7], are presented in this sub section while all NMR spectra of PILs (Figures S1–S12) are available in the Supplementary Materials. The reported water content for all PILs is between 1.04% and 8.70%. Based on reported data by Chen et al., PILs are highly hygroscopic and own higher hydrophilicity in comparison to aprotic ionic liquids [38]. Meanwhile, the presence of water molecules lowers the electrostatic attractions between the ions and consequently reduces the viscosity of ILs [39]. Nonetheless, the thermophysical properties of our PILs are solely reported by using these water content values.

[EHA][C5]: ^1^H NMR (500 MHz, CDCl_3_): δ 0.902 [t, 9H (R-CH_3_)], δ 1.518 [m, 13H (R-CH, R-CH_2_), δ 2.120 [t, 2H (COOH-CH_2_)], δ 2.710 [m, 2H (NH_2_-CH_2_)]. ^13^C NMR (125 MHz, CDCl_3_): δ 181.05, 42.48, 37.97, 37.82, 30.17, 28.66, 28.43, 23.22, 22.88, 22.76, 13.98, 13.91, 10.14. Water content: 6.37%.

[EHA][C6]: ^1^H NMR (500 MHz, CDCl_3_): δ 0.880 [t, 9H (R-CH_3_)], δ 1.516 [m, 15H (R-CH, R-CH_2_), δ 2.118 [t, 2H (COOH-CH_2_)], δ 2.690 [m, 2H (NH_2_-CH_2_)]. ^13^C NMR (125 MHz, CDCl_3_): δ 181.22, 42.61, 38.09, 37.97, 31.92, 30.20, 28.14, 23.22, 22.89, 22.54, 13.97, 13.88, 10.16. Water content: 8.70%.

[EHA][C7]: ^1^H NMR (500 MHz, CDCl_3_ δ 0.900 [t, 9H (R-CH_3_)], δ 1.508 [m, 17H (R-CH, R-CH_2_), δ 2.086 [t, 2H (COOH-CH_2_)], δ 2.688 [m, 2H (NH_2_-CH_2_)]. ^13^C NMR (125 MHz, CDCl_3_): δ 181.11, 42.51, 38.22, 38.02, 31.79, 30.19, 29.45, 28.44, 26.55, 23.21, 22.90, 22.58, 14.00, 13.97, 10.14. Water content: 8.43%.

[BEHA][C5]: ^1^H NMR (500 MHz, CDCl_3_): δ 0.836 [m, 15H (R-CH_3_)], 1.265 [m, 16H (R-CH_2_, -CH)], δ 1.523 [m, 4H (R-CH_2_)], δ 2.149 [t, 2H (CH_2_-COO-)], δ 2.576 [d, 4H (CH_2_-NH)]. ^13^C NMR (125 MHz, CDCl_3_): δ 179.31, 51.75, 37.15, 36.37, 30.80, 28.53, 28.02, 23.95, 22.96, 22.57, 14.02, 13.86, 10.38. Water content: 1.52%.

[BEHA][C6]: ^1^H NMR (500 MHz, CDCl_3_): δ 0.811 [m, 15H (R-CH_3_)], δ 1.282 [m, 18H (R-CH_2_, -CH)], δ 1.534 [m, 4H (R-CH_2_)], δ 2.124 [t, 2H (CH_2_-COO-)], δ 2.585 [d, 4H (CH_2_-NH)]. ^13^C NMR (125 MHz, CDCl_3_): δ 179.25, 51.74, 37.15, 36.71, 31.74, 30.79, 28.51, 25.63, 23.94, 22.96, 22.50, 14.01, 13.96, 10.35. Water content: 1.04%.

[BEHA][C7]: ^1^H NMR (500 MHz, CDCl_3_): δ 0.865 [m, 15H (R-CH_3_)], δ 1.280 [m, 20H (R-CH_2_, -CH)], δ 1.540 [m, 4H (R-CH_2_)], δ 2.126 [t, 2H (CH_2_-COO-)], δ 2.582 [d, 4H (CH_2_-NH)]. ^13^C NMR (125 MHz, CDCl_3_): δ 179.29, 51.65, 37.00, 36.85, 31.67, 30.74, 29.20, 28.48, 28.47, 25.93, 23.90, 22.92, 22.53, 13.96, 10.27. Water content: 1.37%.

### 2.2. TGA and DSC Analysis

**Figure 1:**
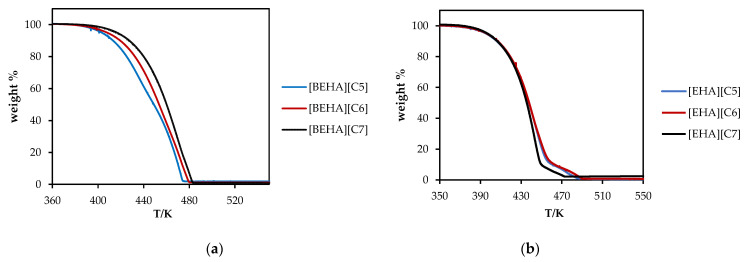
Thermal decomposition curves of (**a**) [EHA][C5], [EHA][C6], [EHA][C7], and (**b**) [BEHA][C5], [BEHA][C6], [BEHA][C7] at heating rate of 10 °C.min^−1^.

Thermogravimetric analyzer (TGA) was used to study the thermal stability of the PILs. Table 1 shows the thermal stability data while Figure 1 displays the TGA profiles of the synthesized PILs. It could be observed from the data that the thermal stability of PILs is in the range of 416 to 437 K. For a common cation, lengthening the alkyl chain branch in the anion caused an increment in the thermal decomposition (T_d_) of the PIL. This could be evidenced by the relatively high T_d_ of [EHA][C7] and [BEHA][C7] as compared to the others. A similar observation was reported by Bhattacharyya et al. in which the thermal stability of an amino acid ionic liquid with longer alkyl chain attached to the nitrogen, [N_1_,_1_,_14_,_2O12_][Lys], has a higher thermal stability than another amino acid ionic liquid, [N_1_,_1_,_6_,_2O12_][Lys], with a relatively shorter alkyl chain branch [40]. According to Keshapolla et al. and Bandres et al., the relationship between high thermal stability and long alkyl chain attached to the ionic liquid could be attributed to the presence of strong intermolecular and intramolecular forces in the alkyl chain [41,42]. In addition, it was observed that the T_d_ values of PILs with a common cation are relatively close to one another and a similar observation was recorded by Cai et al., involving a series of ionic liquids namely triethanolamine methanesulfonate [TEA][mesy], triethanolamine trifluoromethanesulfonate [TEA][OTf] and triethanolamine benzenesulfonate [TEA][Bsa] [43]. On the other hand, the thermal stability of the ammonium-based PILs synthesized in this work is relatively low when compared to other types of ionic liquids. For instances, the thermal stability of an imidazolium-based ionic liquid (1-butylimidazolium dicyanamide, [BMIM] [DCA]) and a phosphonium-based ionic liquid, (phosphonium bis-dicarbollylcobalt (III) [PC6C6C6C14][CoCB]) are greater than 300 °C [35,44,45]. Xu and Cheng have summarized that the thermal stability of imidazolium ionic liquids was improved by increasing the degree of substitution of hydrogen by alkyl groups on the imidazolium ring [46].

**Figure 2:**
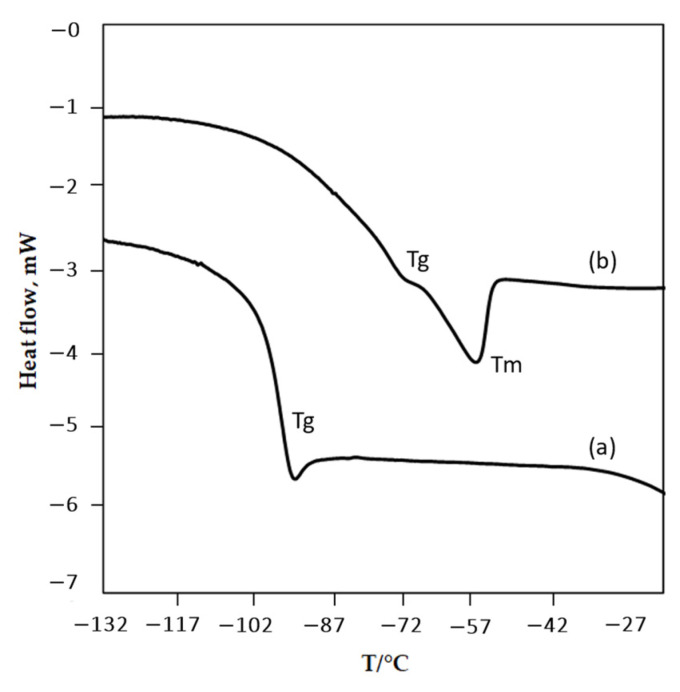
Differential scanning calorimetry (DSC) curves of (a) [EHA][C7], and (b) [BEHA][C6] at a heating rate of 10 °C min^−1^.

The phase transitions which are glass transition temperature (T_g_), and melting point (T_m_) of the ammonium-based PILs were investigated by using a Differential Scanning Calorimeter (DSC) from −150 °C to 50 °C and the results are tabulated in Table 1. This temperature range was chosen based on the fact that many ILs exhibit glass transition at low temperatures even beyond −100 °C [47]. Apart from providing the fundamental information, the study of phase transition of PILs at this condition is crucial due to demand in other technological areas with extreme environments. For example, in space-related applications, ILs is potentially being used as hypergolic fluids in orbiting satellites, manned spacecraft and deep-space probes [48]. Figure 2 shows the examples of DSC curves for the ammonium-based PILs synthesized in this study. Data show that all PILs possess a glass transition temperature (T_g_) ranging from −98.37 °C to −90.89 °C, which indicates that all PILs experience the flow of heat from amorphous glass to liquid state [19]. As T_g_ represents the cohesive energy of the sample, PILs that exhibit T_g_ values have low cohesive energy that could contribute to advantageous physiochemical properties such as low viscosity and high ionic conductivity [47]. A similar trend of marginal difference in the T_g_ values for the ammonium-based PILs was also observed and discussed by other researchers employing ammonium-based ionic liquids as well [47]. In contrast, only ammonium-based PILs with [BEHA] cation exhibited a melting temperature (T_m_) in which all T_m_ are in the range of −68.34 °C to −66.69 °C. Only a minimal increment in the T_m_ values was observed when the alkyl chain of anion increases [C5] to [C7]. Primarily, the T_m_ of PIL is dependent on the crystal lattice strength in the PIL. The low T_m_ of the PIL could be related to the low crystal lattice energy due to poor packing efficiency in the crystal lattice of PIL itself [43,49]. The data obtained in this work suggests that [BEHA][C7] has a better packing of the counterions in its structure than that of [BEHA][C5] and [BEHA][C6].

### 2.3. Density (ρ), Thermal Expansion Coefficient (α_p)_, Standard Entropy (S°) and Lattice Potential Energy (U_pot_) Measurement

**Figure 3:**
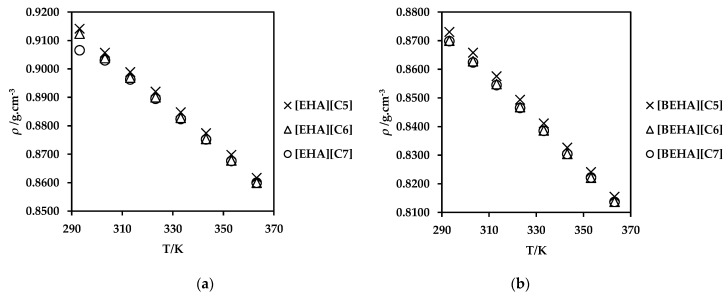
Density (*ρ*) values of (**a**) [EHA][C5], [EHA][C6], [EHA][C7], and (**b**) [BEHA][C5], [BEHA][C6], [BEHA][C7] as a function of temperature.

The density of ammonium-based PILs was studied at temperatures ranging from 293.15 to 363.15 K. The plots of the experimental density of the PILs are shown in Figure 3 while the experimental data and the plots with standard errors are available in Table S1 and Figure S13, respectively, in the Supplementary Material. As illustrated in Figure 3, the densities for all six ammonium-based PILs decreased linearly with temperature. Experimental data also indicates that the density of the PILs deceases as the alkyl chain of the anion increases for both [EHA] and [BEHA] PILs. The results are in accordance with published results in literature for PILs with diethylammonium and dibutylammonium cations with the density values ranging approximately from 0.82 g.cm^−3^ to 0.94 g.cm^−3^ [50]. A similar observation was also found by other researchers when the densities of their tetrabutylammonium ionic liquids were analyzed over a temperature range of 283.4 to 333.4 K [51]. As temperature increases, the volume of ionic liquids increases, and the density of the ionic liquids decreases accordingly. At higher temperatures, the intermolecular forces between the constituent ions weaken, and this increases the mobility of the ions which in turn increases the volume of these ions [37,52,53]. Further analysis also revealed that [EHA][C5] has the highest density values compared to the rest of the ammonium-based PILs. The small size of [EHA] cation compared to [BEHA] cation affects local packing of the PIL structure and thus contributes to the increase in the density values of [EHA][C5] [37,54]. Comparable observations using PIL with ethylammonium cation were also found by several researchers, in which an increasing trending packing efficiency was proportional with the decreasing of molecular weight [51,55]. Notably, the increased alkyl chain length in both cation and anion of the PIL has promoted the steric hindrance and asymmetric nature in the PIL structure as bigger and bulkier PILs result in a lower density value for the PILs [40,41]. This trend can be observed in [BEHA][C6] and [BEHA][C7] as they exhibit the lowest density values.

The thermal expansion coefficient can provide information about the intermolecular interaction in the PILs, and it can be calculated from the experimental values of density, *ρ* by using Equation (1). The calculated data is tabulated in Table 2. Thermal expansion coefficients, *α_p_* for the ammonium-based PILs can be defined as [37,53,56]:

*α_p_* = −1/*ρ*. (δ*ρ*/δT) = −(A_2_)/(A_1_ + A_2_T) (1)

The calculated values in Table 2 show that the thermal expansion coefficients vary only slightly with the increase of C-numbers in the structure of the PILs. PILs with [BEHA] cation has higher *α_p_* than that of PILs with [EHA] cation. This indicates that the thermal expansion coefficient does not only depend on the cation symmetry but is also related to the length of the alkyl substituent [57]. Meanwhile, the behavior of the thermal expansion coefficient is almost similar for all PILs with common cations. Sarkar et al. have also reported a similar variation trend of the thermal expansion coefficient for diethylammonium-based PILs [19]. To conclude, the thermal expansion coefficient can be considered as temperature independent as it shows similar results over the temperature range studied.

The volume occupied by one mole of a compound at a given temperature and pressure is denoted as molar volume, V_m_. The molar volume was calculated by using an empirical equation as shown in Equation (2) and utilizing the experimental densities [41,58,59,60,61]:

V_m_ = M/(*ρ*. N_A_) (2)

where V_m_ is the molar volume, M is the molar mass of the ammonium-based PILs, *ρ* is the density of PILs at 303.15 K and N_A_ is Avogadro’s number.

The calculated molar volume for all ammonium-based PILs are tabulated in Table 3. From the calculated value, the molar volume, V_m_, is proportional to the anion alkyl chain length as well as the size of the cation. The molar volume increases with the alkyl chain length of the anion and this behavior is caused by the addition of the CH_2_ group in the anion of the PILs. Besides that, PILs with [BEHA] cation exhibit a larger molar volume value compared to PILs with [EHA] cation. This could be explained by the difference in the size of the cations. Similar findings have been observed in other studies [19,37].

Entropy is the measurement of the randomness of molecules, and generally, entropy increases with molar volume [19]. The relationship between molar volume (V_m_) and standard entropy (S°) for the ammonium-based PILs in this work can be explored by using the following standard equation that is available in the literature [62]:

S° = 1246.5 V_m_ + 29.5 (3)

The results presented in Table 3 clearly indicate that the standard entropy increased with the molar volume value for all ammonium-based PILs. The increasing number of carbon atoms in the alkyl chain of carboxylate anion has resulted in the increment of the S° of the ammonium-based PILs. From the calculated values obtained, [BEHA]-based PILs depicted the highest standard entropy due to their larger size compared to [EHA]-based PILs, which causes the least interaction between cation and anion [41]. In this work, the standard entropy of [EHA] and [BEHA] PILs increases in the sequence of [C5] < [C6] < [C7].

In addition, to predict the relative stabilities of ILs, Glasser [62] has also developed a method for calculating lattice potential energies (U_pot_) of ILs by using Equation (4):

U_pot_ = [γ (*ρ*/M)^1/3^] + δ (4)

where γ and δ are fitting coefficients with values of 1981.9 kJ·mol^−1^ and 103.8 kJ·mol^−1^, respectively.

The lattice potential energy of the studied PILs was calculated at 303.15 K. The main factor contributing to lattice potential energy is electrostatic or columbic interaction. However, lattice potential energy is inversely related to the volume of ions [19,52,54]. As can be seen in Table 3, lattice potential energy decreases with the addition of the carbon chain length of the carboxylate groups. The addition of methylene group in the alkyl chain of both cation and anion increases the entropy, and consequently reduces packing efficiency in the PILs [63]. As a result, lattice potential energy will decrease with the increase in the alkyl chain length of the PILs.

### 2.4. Viscosity (η) Measurement

**Figure 4:**
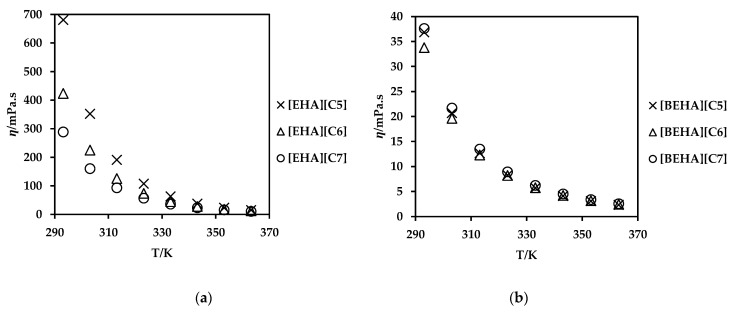
Viscosity (ŋ) values of (**a**) [EHA][C5], [EHA][C6], [EHA][C7], and (**b**) [BEHA][C5], [BEHA][C6], [BEHA][C7] as a function of temperature.

Viscosity is one of the important properties that governs the potential applications of any solvents, and it is largely influenced by intermolecular interactions namely hydrogen bonding, dispersive forces and columbic interactions [64]. The experimental data and the plots with standard errors for viscosity values are available in Table S2 and Figure S14, respectively, in the Supplementary Materials. The viscosity was measured in a temperature range of 293.15 to 363.15 K and graphically shown in Figure 4. The viscosity of all ammonium-based PILs decreased exponentially with an increase in temperature in each PIL as depicted in Figure 4. For example, the viscosity of [EHA][C5] at 293.15K is 45 times larger than at 363.15K. In another study, Liu et al. performed dynamic density measurement on three series of ILs, namely N-alkylpyridinium bis(trifluoromethylsulfonyl)imide ([Cnpy][NTf2], *n* = 2, 4, 5, 6), N-alkyl-3-methylpyridinium bis(trifluoromethylsulfonyl)imide ([Cn3mpy][NTf2], *n* = 2, 3, 4, 6) and N-alkyl-4-methylpyridinium bis(trifluoromethylsulfonyl)imide ([Cn4mpy][NTf2], *n* = 3, 4, 6) within a temperature range of T = (283.15 to 353.15) K [60]. They suggested that the dynamic viscosity increases with the extension of the alkyl side chain of the cation for the three series of pyridinium-based ILs. However, in this work, PILs with [EHA] cation display a higher viscosity value than PILs with [BEHA] cation. Basically, the van der Waals attraction between the aliphatic alkyl chain affects the viscosity values of the PILs [41,53]. However, the water content of the PILs may also affected the observed viscosity results. Furthermore, PILs with [BEHA] cation displayed a marginal increment in the viscosity values as the alkyl chain length of the anion increased.

### 2.5. Refractive Index (n_D_) Measurement

**Figure 5:**
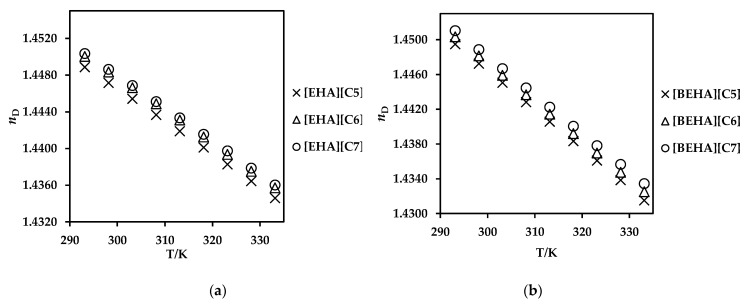
Refractive index (*n*_D_) values of (**a**) [EHA][C5], [EHA][C6] and [EHA][C7], and (**b**) [BEHA][C5], [BEHA][C6] and [BEHA][C7] as a function of temperature.

Generally, the refractive index (*n*_D_) describes how fast light travels through material. It estimates the electronic polarizability of the molecules and shows the dielectric response to an external electric field produced by electromagnetic waves (light) [65]. Figure 5 shows the refractive index of ammonium-based PILs that were measured in a temperature range of 293.15 to 333.15 K at atmospheric pressure. The experimental data is tabulated in Table S3 while the plots with standard errors are presented in Figure S15 in the Supplementary Material. From the table, the *n*_D_ values were found to be decreasing with increasing temperature. Moreover, the values of the refractive index increased with the increase in cation and anion chain length of PILs. A similar observation was also found in the literature involving PILs in which the *n*_D_ values of the studied PILs were in the range of 1.45–1.41 [50]. The increment of refractive index values with increasing alkyl chain length in the cation is influenced by higher intermolecular interaction such as the van der Waals forces of the PILs [52].

### 2.6. Thermophysical Properties Correlations

The density (*ρ*), dynamic viscosity (*η*) and refractive index (*n*_D_) experimental values were correlated by using the following equations [53,66]:

*ρ* = A_1_ + A_2_T (5)

lg*η* = A_3_ + A_4_/T (6)

*n*_D_ = A_5_ + A_6_T (7)

where T is the temperature in K, and A_1_ through A_6_ are correlation coefficients using the least square method. Table 4 represents the estimation of values for correlation coefficients together with the standard deviations, *SD* which was calculated by using the Equation (8). *Z_expt_* and *Z_calc_* are experimental and calculated values, respectively, while *n_DAT_* is the number of experimental points.

(8) $$SD=\sqrt{\frac{\sum_{i}^{n_{DAT}}{\left(Z_{expt}-Z_{calc}\right)}^{2}}{n_{DAT}}}$$

### 2.7. CO_2_ Absorption Measurement

**Figure 6:**
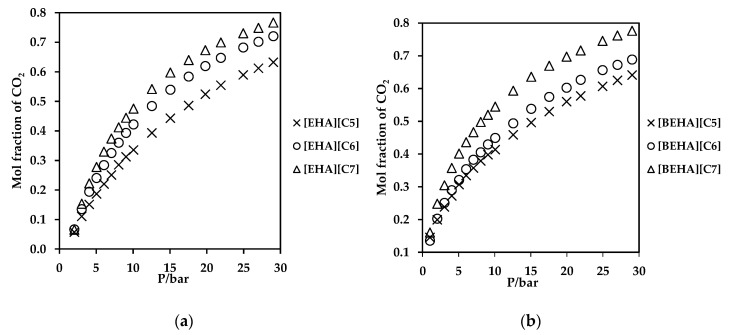
Plot of CO_2_ absorption in ammonium-based PILs with (**a**) [EHA] cation, and (**b**) [BEHA] cation.

**Figure 7:**
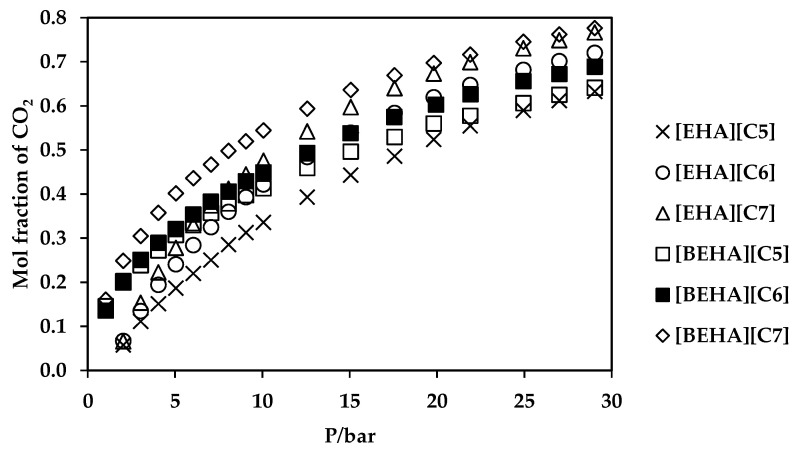
Plot of CO_2_ absorption in ammonium-based PILs at 298.15 K.

Carbon dioxide absorption measurements have been performed to investigate the potential ability of the ammonium-based PILs as solvents for CO_2_ capture. The measurements were conducted in the pressure range of 1-29 bar at room temperature and the results are plotted in Figure 6 and Figure 7. From the plots, the CO_2_ uptake by the ammonium-based PILs shows a trend of polynomial increment with CO_2_ pressure. Generally, ammonium-based PILs with [BEHA] cation exhibited marginal difference in CO_2_ absorption values than that of ammonium-based PILs with [EHA] cations as shown in Figure 7. At a constant pressure of 29 bar, [BEHA][C7] displayed the highest CO_2_ absorption with the value of 0.78 mol fraction when compared to [BEHA][C6] and [BEHA][C5] with the CO_2_ mol fractions of 0.68 and 0.64, respectively. This behavior can be explained by using the data reported of density and molar volume of the ammonium-based PILs. The increment in the density value of the PIL increases the molar volume of the PILs which thus in turn causes an increase in the fractional free volume and consequently enhances the CO_2_ uptake by the ammonium-based PILs [67,68]. Based on the analysis and comparison of FTIR and ^13^C NMR, Xu and Oncsik et al. proposed that the mechanism of CO_2_ absorption is via the interaction between gas and the basic anion [32,69]. At approximately 20 bar and 25 °C, the CO_2_ uptake by the [BEHA][C7] is about 40% higher than that of bis(2-ethylhexyl)ammonium butyrate protic ionic liquid [37]. On the other hand, some researchers have performed investigations on the relationship between the viscosity of PILs and performance of CO_2_ absorption by the PILs and found that PIL with low viscosity value has a high absorption capacity of CO_2_ [70]. ILs with low viscosities can result in low mass transfer resistance between liquid and gas phases, and this eventually increases the CO_2_ absorption rate. The viscosities of [BEHA][C5], [BEHA][C6] and [BEHA][C7] were recorded to have values between 19.64 and 21.70 mPa·s at 30 °C, which are much lower when compared to conventional ILs, for example [Bmim][BF4] with the viscosity value of 68.90 mPa·s at the same temperature [71]. Regardless of the cation, there is an increasing trend of CO_2_ absorption in the order of [C5] < [C6] < [C7] anion. As such, both [EHA][C7] and [BEHA][C7] show the highest CO_2_ absorption capacity at 29 bar and room temperature with the values of CO_2_ mol fractions of 0.77 moles and 0.78 moles, respectively. These results could be considered an indication of the potential ability of the ammonium-based PILs as solvents for CO_2_ capture. However, more thorough studies must be conducted for further evaluation before the ammonium-based PILs can be fully used as new solvents in the field of CO_2_ removal.

## 3. Materials and Methods

### 3.1. Chemicals

To synthesize all six ammonium-based PILs, analytical grade chemicals from Merck, Darmstadt, Germany were used. The CAS numbers, abbreviations, grade percentage, density, viscosity, flash point and melting points of all chemicals are as follows: 2-ethylhexylamine (104-75-6, [EHA], 99.0%, 0.789 g.cm^−3^, 1.1 cP, 140 °C, −76 °C), bis-(2-ethylhexyl)amine (106-20-7, [BEHA], 99.0%, 0.805 g.cm^−3^, 3.7 cP, 130 °C, <−20 ° C), pentanoic acid (109-52-4, [C5], 98.0%, 0.939 g.cm^−3^, 2.3 cP, 96 °C, −34.5 °C), hexanoic acid (142-62-1, [C6], 98.0%, 0.927 g.cm^−3^, 3.2 cP, 102 °C, −2.78 °C) and heptanoic acid (111-14-8, [C7], 99.0%, 0.917 g cm^−3^, 3.4 cP, 113 °C, −7.5 °C)).

### 3.2. Synthesis of PILs

The synthesis of PILs was carried out by using a one-step neutralization reaction and the reaction is written as follows:

(R_x_)_2_NH + HOOC(R_Y_) → (R_x_)_2_NH_2_^+ −^OOC(R_Y_)

R_X_ is 2-ethylhexyl or bis-(2-ethylhexyl and R_Y_ is either pentyl, hexyl or heptyl. In this work, 2-ethylhexylamine and bis-(2-ethylhexyl)amine, abbreviated as [EHA] and [BEHA], respectively, were the cations while the acids with the variation of alkyl chain length from pentyl [C5], hexyl [C6] and heptyl [C7] were the anions of the PILs.

**Figure 8:**
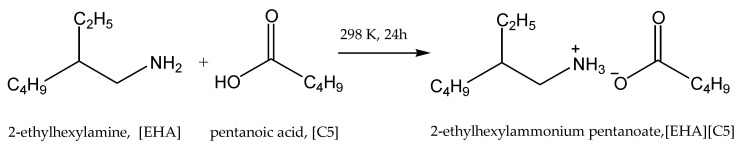
Synthesis reaction for [EHA][C5].

In a specific procedure, a 1:1 mol ratio of acid was added to the base with continuous stirring at 250 rpm for 24 h at room temperature. The resulting product was dried under vacuum at 80 °C for 6 h to remove any water traces and impurities that might be present resulting from starting reagents as well as surrounding atmosphere. The PILs which were in liquid forms without noticeable solid crystal or precipitation after the purification step were kept in sealed containers until further analysis. The proton transfer reaction had resulted in the formation of six PILs as tabulated in Table 5. Figure 8 depicts the reaction for the synthesis of [EHA][C5].

### 3.3. Characterization

#### 3.3.1. Structural Confirmation and Water Content

Nuclear Magnetic Resonance (NMR) spectroscopy (Bruker Ascend TM 500, Billerica, MA, USA) was used to confirm the structures of the synthesized PILs. In each analysis, a 100 μL PIL sample was dissolved in a 600 μL solvent (CDCl_3_). The ^1^H and ^13^C spectra are reported in parts per million and the multiplicities, where applicable, are written as *d* (doublet), *t* (triplet) and *m* (multiplet). The water content of each PIL was determined by using Volumetric Karl Fisher V30 Mettler Toledo (Columbus, OH, USA).

#### 3.3.2. TGA Analysis

A Simultaneous Thermal Analyzer (STA) 6000 (Perkin Elmer, Waltham, MA, USA) was used to study the thermal stability of the PILs. The reproducibility for TGA STA 6000 is <±0.5 °C with ± 2% based on metal standard. In each analysis, 10 mg of the PILs sample was placed in a crucible pan and the thermal analysis was conducted in a temperature range of 50–650 °C at a heating rate of 10 °C·min^−1^ under 20 mL/min of nitrogen flow.

#### 3.3.3. DSC Analysis

A Differential Scanning Calorimeter (DSC) 1 Star system (Mettler Toledo, Columbus, OH, USA) was used to investigate the phase transition of the PILs. The reproducibility for DSC is ± 0.2K with <1% based on indium calibration. In total, 10 mg of samples were sealed in aluminum pans and subject to analysis in a temperature range of 50–150 °C with a heating rate of 10 °C·min^−1^. The phase transition data were analyzed by using the second heating plot.

#### 3.3.4. Density (*p*) and Viscosity (*ŋ*) Measurement

An Anton Paar Stabinger Viscometer (Graz, Austria) was used to simultaneously measure the density and viscosity of the PILs in a temperature range of 293.15–363.15 K with a temperature measurement accuracy of 0.02 K. The reproducibility of the density and viscosity measurements were ±5.10^−4^ g.cm^−3^ and 0.35%, respectively. The measurements were repeated several times and the average value was considered for further analysis. Prior to the density and viscosity measurements, the equipment was calibrated using a standard fluid provided by the supplier. A commercial imidazolium IL with known density and viscosity values was also used to validate the equipment.

#### 3.3.5. Refractive Index (*n_D_*) Measurement

An ATAGO RX-5000 Alpha Digital Refractometer (Tokyo, Japan) with a measuring accuracy of ±4.10^−5^ was used to determine the refractive index values of the PILs. The measurement was done in a temperature range of 293.15 to 333.15 K. The instrument was also calibrated by using standard organic solvents provided by the supplier. In addition, a commercial imidazolium IL was also used while conducting the validation test and the result was compared with the values available from the literature [50].

#### 3.3.6. CO_2_ Absorption Measurement

The CO_2_ absorption of the PILs was studied by using a magnetic suspension balance (MSB) from Rubotherm Präzisionsmesstechnik GmbH (Bochum, Germany). In this gravimetric method, the weight change of the PILs upon absorption of CO_2_ was measured and calculated in a range of pressure from 1 to 29 bar at room temperature. The sample absorption chamber linked to the microbalance, which has a precision of ±20 μg, via an electromagnet and a suspension magnet which keeps the balance at ambient conditions during the CO_2_ absorption experiments. In a typical CO_2_ absorption measurement, approximately 1g of PIL sample was loaded in the sample chamber and the absorption system was evacuated at 10^−3^ mbar (Pfeiffer model DUO5) to remove any impurities until the weight remained constant. Then, the sample chamber was pressurized with CO_2_ at a constant temperature by means of an oil circulator (Julabo, model F25-ME, ±0.1 °C accuracy, Seelbach, Germany) and the weight change due to the absorption of the gas in the PIL was observed and recorded. Once a constant weight reading was recorded, the system was allowed to stand in the condition for an additional 3-4 h to ensure complete equilibration of the binary CO_2_–PIL system. The absorption measurement was repeated with different pressure values of CO_2_ to yield a series of absorption isotherm. The weight of the CO_2_ dissolved in the PILs sample was calculated using Equation (9) available from literature [72,73].

wt CO_2_= [wt − (wt_Sc_ + wt_S_)] + [(V_Sc_ + V_S_)(*ρ*CO_2_)] (9)

where wt (g) is the corrected weight of the balance, wt_Sc_ + wt_S_ (g) are the weights of sample cell and sample, respectively, V_Sc_ + V_S_ (cm^−3^) are the volumes of the sample cell and sample, respectively, and *ρ*CO_2_ (g.cm^−3^) is the density of CO_2_ at the pressure and temperature during the CO_2_ absorption. The results of CO_2_ absorption are presented in terms of mole fraction of CO_2_ (x) dissolved in the PIL, which was calculated using Equation (10):

x = n_CO2_/(n_liq_ +n_CO2_) (10)

where n_CO2_ is the mole of CO_2_ absorbed in the PIL and n_liq_ is the mole of the PIL.

## 4. Conclusions

In this work, six new ammonium-based PILs have been successfully synthesized through a one-step procedure. The thermophysical properties including density, viscosity, refractive index and thermal stability have been measured. The experimental results revealed the dependency of the experimental values namely the *ρ*, *η*, *n*_D_ and T_d_ on the alkyl chain of the anion, size of the cations and the temperature of measurement. The phase transition analysis of the PILs yielded the glass transition temperature (T_g_) and melting point (T_m_) of the PILs studied. These synthesized ammonium-based PILs have been tested for their ability towards CO_2_ absorption in which [BEHA][C7] displayed the highest CO_2_ uptake in the experimental conditions signifying its capability to be a potential solvent in the application of CO_2_ capture. Future works should include CO_2_ desorption studies of the PILs for the purpose of recyclability and sustainability of the absorbents.

## Figures and Tables

**Table 1:** Thermal decomposition, T_d_; glass transition, T_g_; melting point, T_m_.

Ionic Liquids	T_d_	T_g_	T_m_
	K	°C	°C
[EHA][C5]	416.46	−97.00	-
[EHA][C6]	421.85	−98.37	-
[EHA][C7]	424.28	−96.91	-
[BEHA][C5]	428.47	−96.95	−68.34
[BEHA][C6]	431.55	−91.43	−66.82
[BEHA][C7]	437.47	−90.89	−66.69

**Table 2:** Thermal expansion coefficients (*α_p_*) of the PILs calculated using Equation (1).

T/K	10^−4^ α/K^−1^					
	[EHA][C5]	[EHA][C6]	[EHA][C7]	[BEHA][C5]	[BEHA][C6]	[BEHA][C7]
293.15	7.92	7.93	7.79	9.55	9.36	9.27
303.15	7.98	7.99	7.86	9.64	9.45	9.36
313.15	8.05	8.05	7.92	9.73	9.54	9.45
323.15	8.11	8.12	7.95	9.83	9.63	9.54
333.15	8.18	8.18	8.05	9.92	9.72	9.63
343.15	8.25	8.25	8.11	10.02	9.82	9.72
353.15	8.31	8.32	8.18	10.13	9.91	9.82
363.15	8.38	8.39	8.24	10.23	10.01	9.92

**Table 3:** Molar volume, V_m_; standard entropy, S°; lattice potential energy, U_pot_ at 303.15 K.

Ionic Liquids	V_m_	S°	U_pot_
	nm^3^	J·K^−1^·mol^−1^	kJ·mol^−1^
[EHA][C5]	0.4242	558.3	416.0
[EHA][C6]	0.4509	591.5	409.8
[EHA][C7]	0.4771	624.2	404.1
[BEHA][C5]	0.6590	850.9	373.4
[BEHA][C6]	0.6883	887.4	369.5
[BEHA][C7]	0.7156	921.5	366.1

**Table 4:** Fitting parameters of Equation (5) to correlate density (*ρ*) of PILs and calculated standard deviation (SD_1_). Fitting parameters of Equation (6) to correlate viscosity (ŋ) of PILs and calculated standard deviation (SD_2_). Fitting parameters of Equation (7) to correlate refractive index (*n*_D_) of PILs and calculated standard deviation (SD_3_).

ILs	A_1_	A_2_	SD_1_	A_3_	A_4_	SD_2_	A_5_	A_6_	SD_3_
[EHA][C5]	1.1297	−0.0007	0.0043	2842.7	−8.1026	0.002	1.5535	−0.0004	0.00005
[EHA][C6]	1.1279	−0.0007	0.0004	1772.7	−5.0403	0.006	1.5555	−0.0004	0.00118
[EHA][C7]	1.1091	−0.0007	0.0012	1217.3	−3.4491	0.006	1.5553	−0.0004	0.00146
[BEHA][C5]	1.1161	−0.0008	0.0004	152.1	−0.4278	0.027	1.5809	−0.0004	0.00146
[BEHA][C6]	1.1076	−0.0008	0.0004	139.8	−0.3918	0.024	1.5810	−0.0004	0.00103
[BEHA][C7]	1.1056	−0.0008	0.0003	155.6	−0.4367	0.024	1.5802	−0.0004	0.00115

**Table 5:** Structures of acids and bases, their names, and abbreviations used.

Structure	Name and Abbreviation
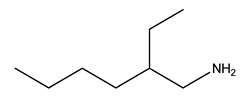	2-ethylhexylamine, [EHA]
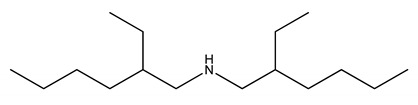	Bis-(2-ethylhexyl)amine, [BEHA]
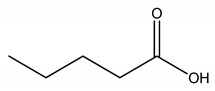	Pentanoic acid, [C5]
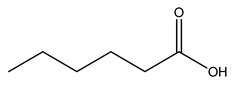	Hexanoic acid, [C6]
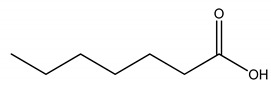	Heptanoic acid, [C7]

## Data Availability

The data presented in this study is available in this article and Supplementary Materials.

## Supplementary Materials

The following are available online, Table S1: Density (*ρ*) values of PILs at temperatures (293.15–363.15) K, Table S2: Dynamic viscosity (*η*) values of PILs at temperatures (293.15–363.15) K, Table S3: Refractive index (*n*_D_) values of PILs at temperatures (293.15–333.15 K), Figures S1–S12: NMR analysis of the PILs, Figure S13: Plots of density (*ρ*) values with standard errors of (a) [EHA][C5], [EHA][C6], [EHA][C7], and (b) [BEHA][C5], [BEHA][C6], [BEHA][C7] as a function of temperature, Figure S14: Plots of viscosity (*η*) values with standard errors of (a) [EHA][C5], [EHA][C6], [EHA][C7], and (b) [BEHA][C5], [BEHA][C6], [BEHA][C7] as a function of temperature, and Figure S15: Plots of refractive index (*n*_D_) values with standard errors of (a) [EHA][C5], [EHA][C6], [EHA][C7], and (b) [BEHA][C5], [BEHA][C6], [BEHA][C7] as a function of temperature.
