# Supplementary material for: Experimental Investigation on Thermophysical Properties of Ammonium-Based Protic Ionic Liquids and Their Potential Ability towards CO2 Capture
Source: Molecules. 2022 Jan 27;27(3):851. doi: 10.3390/molecules27030851 (PMC8839255; doi:10.3390/molecules27030851)
Supplement: Supplementary file 1 [file molecules-27-00851-s001.zip › molecules-1558613-supplementary.pdf]

SUPPLEMENTARY MATERIALS

Experimental investigation on thermophysical properties of ammonium-based protic ionic liquids and their potential ability towards CO_2 capture

Nur Hidayah Zulaikha Othman Zailani [1], Normawati M. Yunus [1*], Asyraf Hanim Ab Rahim [1] and Mohamad Azmi Bustam [2]

[1] Department of Fundamental and Applied Sciences, Centre of Research in Ionic Liquids (CORIL), Institute of Contaminant Management for Oil and Gas, Universiti Teknologi PETRONAS, Seri Iskandar 32610, Malaysia; zulaikha95.nhz@gmail.com (N.H.Z.O.Z.); asyrafhanim92@gmail.com (A.H.A.R.)

[2] Department of Chemical Engineering, Centre of Research in Ionic Liquids (CORIL), Institute of Contaminant Management for Oil and Gas, Universiti Teknologi PETRONAS, Seri Iskandar 32610, Malaysia; azmibustam@utp.edu.my

* Correspondence: normaw@utp.edu.my; Tel.: +605-368-7689

Table S1. Density (ρ) values of PILs at temperatures (293.15 to 363.15)K.

T/K	ρ /g.cm^{-3}					
	**[EHA][C5]**	**[EHA][C6]**	**[EHA][C7]**	**[BEHA][C5]**	**[BEHA][C6]**	**[BEHA][C7]**
293.15	0.9142	0.9124	0.9066	0.8730	0.8700	0.8698
303.15	0.9057	0.9038	0.9030	0.8658	0.8628	0.8624
313.15	0.8989	0.8969	0.8963	0.8576	0.8548	0.8545
323.15	0.8920	0.8900	0.8895	0.8494	0.8468	0.8465
333.15	0.8848	0.8828	0.8824	0.8411	0.8386	0.8385
343.15	0.8774	0.8754	0.8752	0.8327	0.8304	0.8304
353.15	0.8698	0.8678	0.8676	0.8241	0.8221	0.8222
363.15	0.8617	0.8599	0.8600	0.8155	0.8137	0.8137

Table S2. Dynamic viscosity (η) values of PILs at temperatures (293.15 to 363.15)K.

T/K	η/mPa.s					
	**[EHA][C5]**	**[EHA][C6]**	**[EHA][C7]**	**[BEHA][C5]**	**[BEHA][C6]**	**[BEHA][C7]**
293.15	680.4233	423.3533	288.4767	36.8410	33.7660	37.6123
303.15	352.1600	224.9400	160.4500	20.6940	19.6410	21.7010
313.15	190.8800	125.6700	93.2350	12.6620	12.3160	13.5100
323.15	107.7000	73.3750	56.6750	8.2857	8.2157	8.9414
333.15	62.9150	44.6100	35.8190	5.7253	5.7551	6.2314
343.15	37.8840	28.0490	23.3860	4.1309	4.2028	4.5225
353.15	23.4600	18.1460	15.6460	3.0845	3.1636	3.3859
363.15	14.9000	12.0040	10.6770	2.3314	2.4396	2.5752

Table S3: Refractive index (n_D) values of PILs at temperatures (293.15K to 333.15)K.

T/K	n_D					
	[EHA][C5]	[EHA][C6]	[EHA][C7]	[BEHA][C5]	[BEHA][C6]	[BEHA][C7]
293.15	1.4489	1.4500	1.4503	1.4495	1.4503	1.4510
298.15	1.4472	1.4483	1.4486	1.4473	1.4481	1.4489
303.15	1.4454	1.4466	1.4469	1.4451	1.4459	1.4467
308.15	1.4437	1.4449	1.4451	1.4428	1.4437	1.4445
313.15	1.4419	1.4431	1.4433	1.4406	1.4414	1.4422
318.15	1.4401	1.4413	1.4415	1.4383	1.4392	1.4400
323.15	1.4383	1.4394	1.4397	1.4361	1.4370	1.4378
328.15	1.4364	1.4375	1.4379	1.4339	1.4347	1.4357
333.15	1.4346	1.4357	1.4360	1.4315	1.4325	1.4334

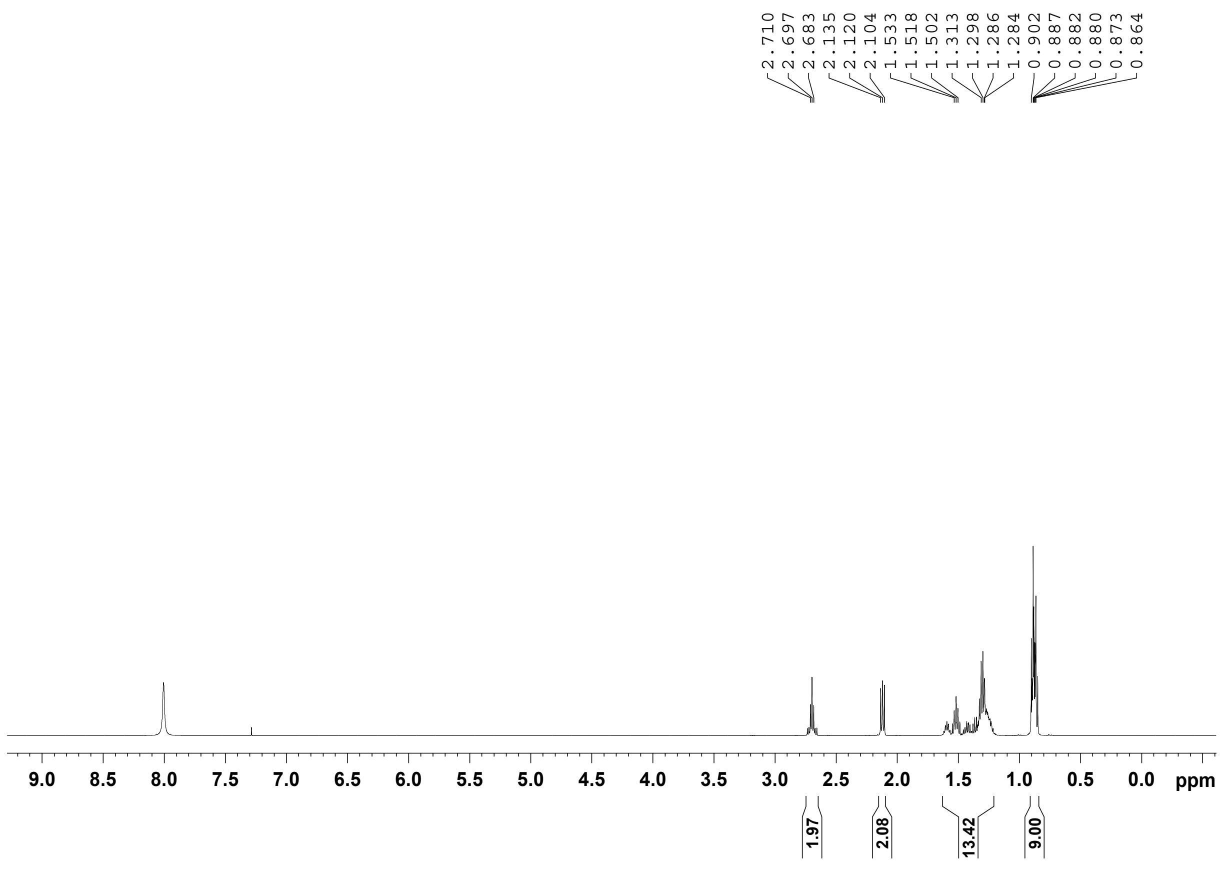

Figure S1: ^{1}H NMR analysis of [EHA][C5]

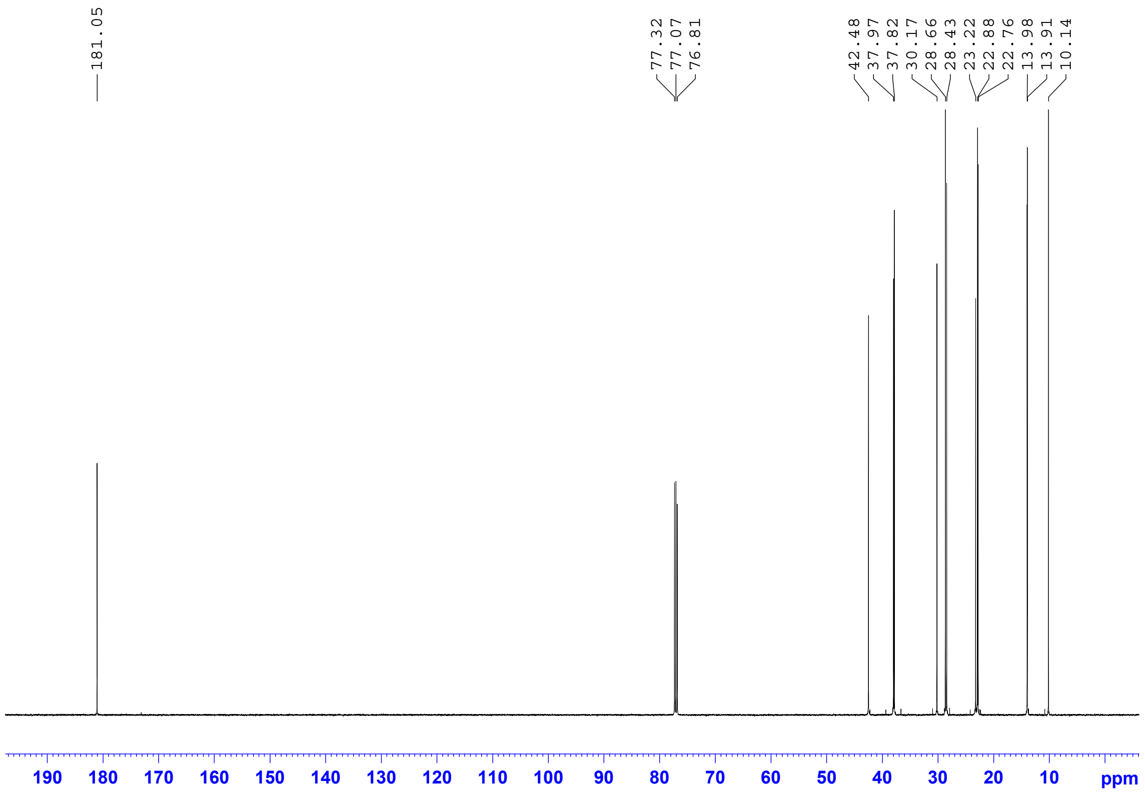

Figure S2: ^{13}C NMR analysis of [EHA][C5]

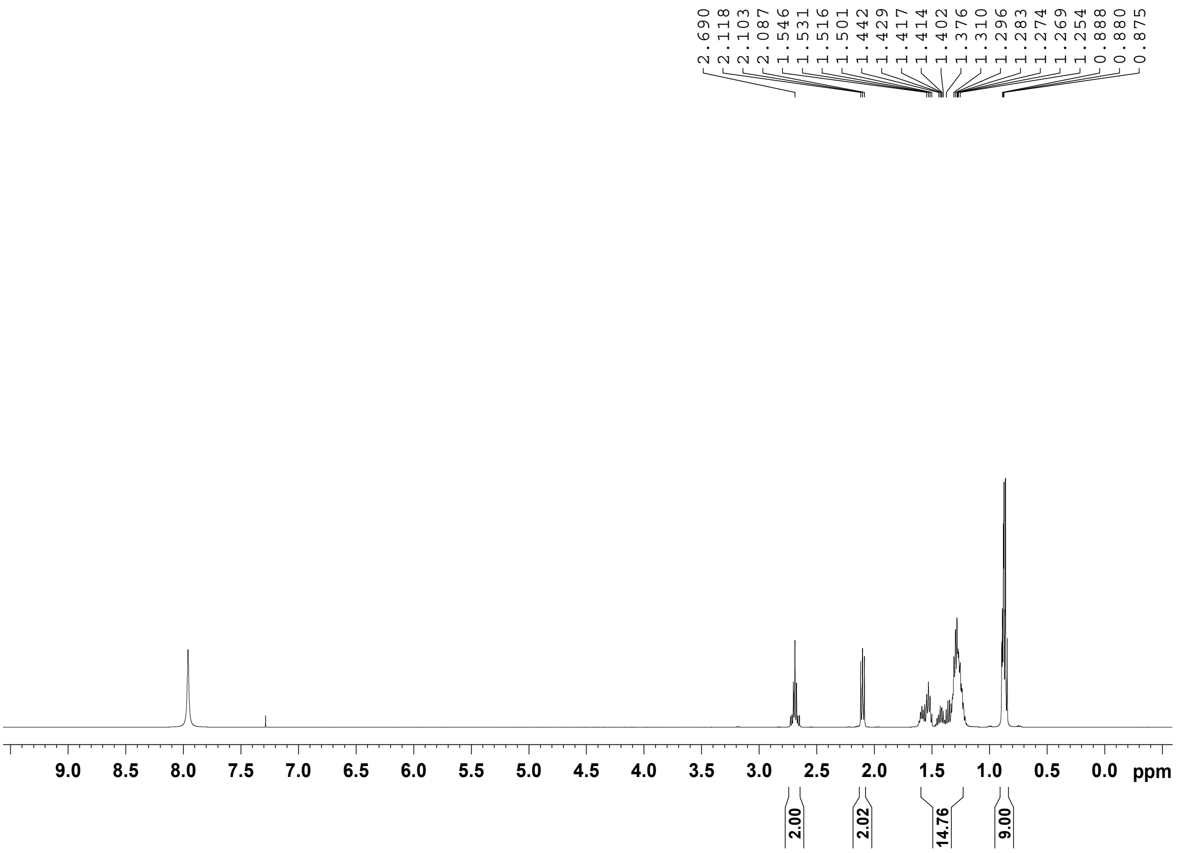

Figure S3: ^{1}H NMR analysis of [EHA][C6]

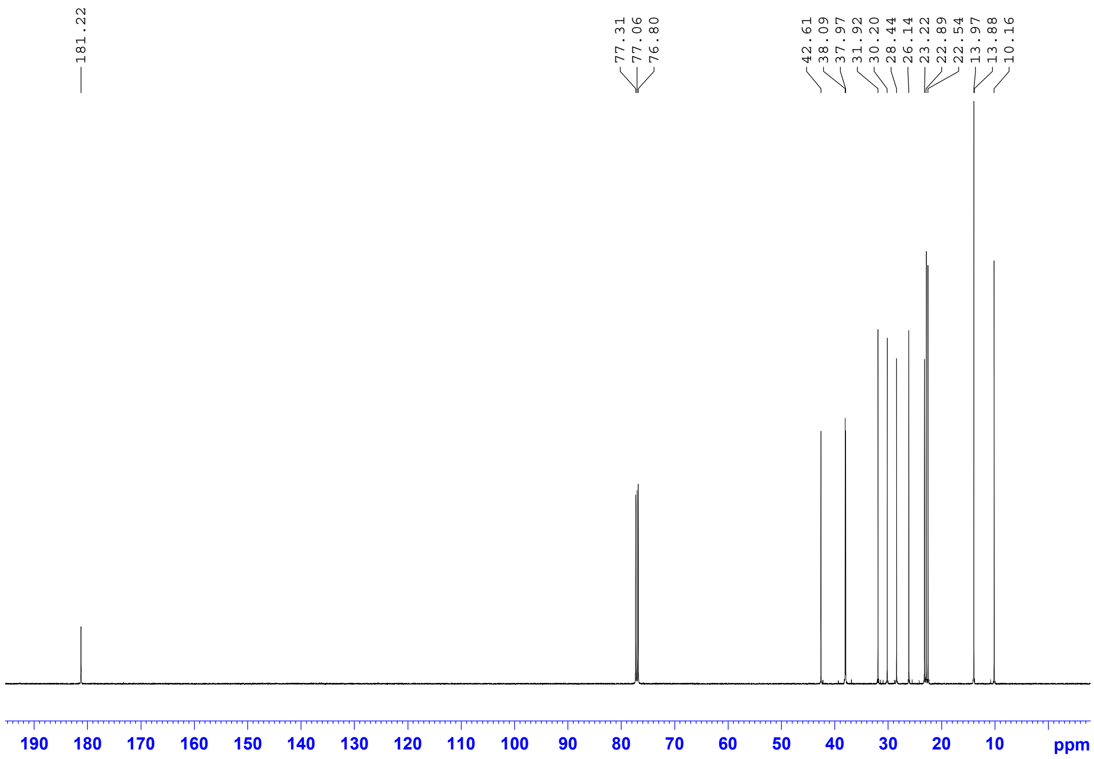

Figure S4: ^{13}C NMR analysis of [EHA][C6]

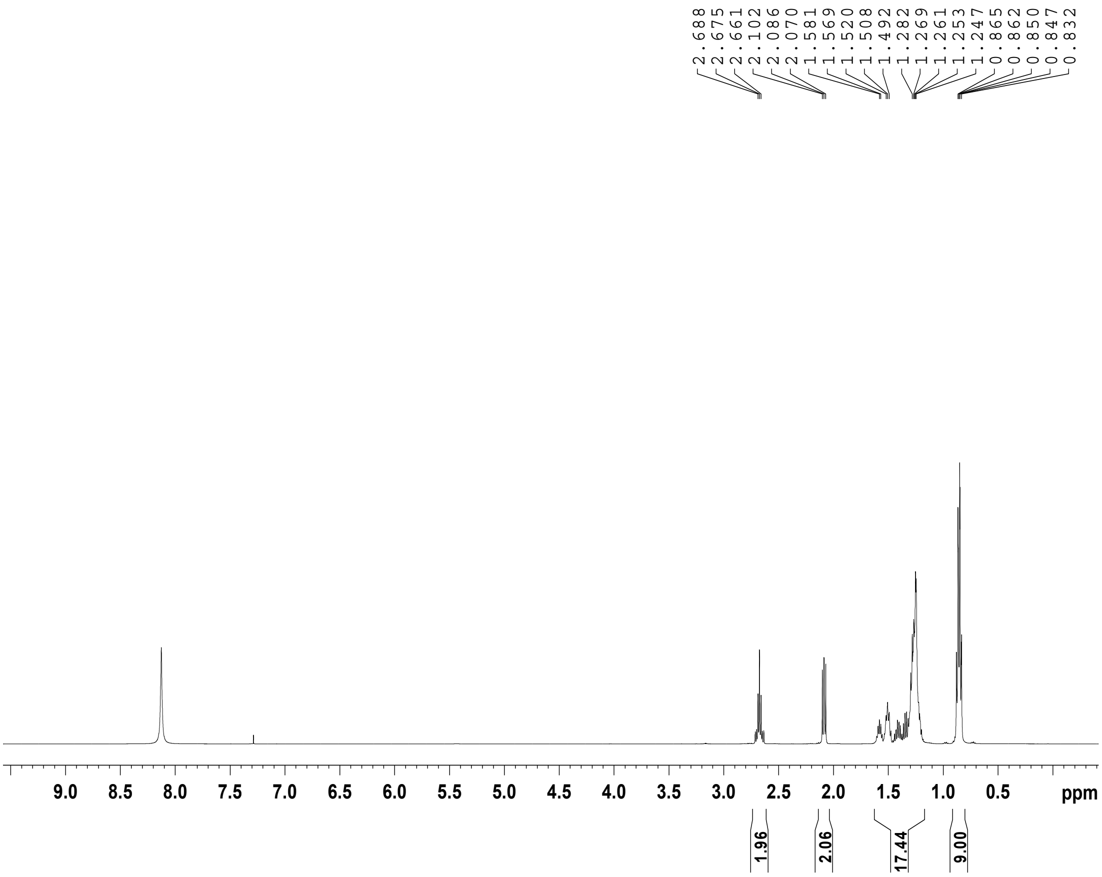

Figure S5: ^{1}H NMR analysis of [EHA][C7]

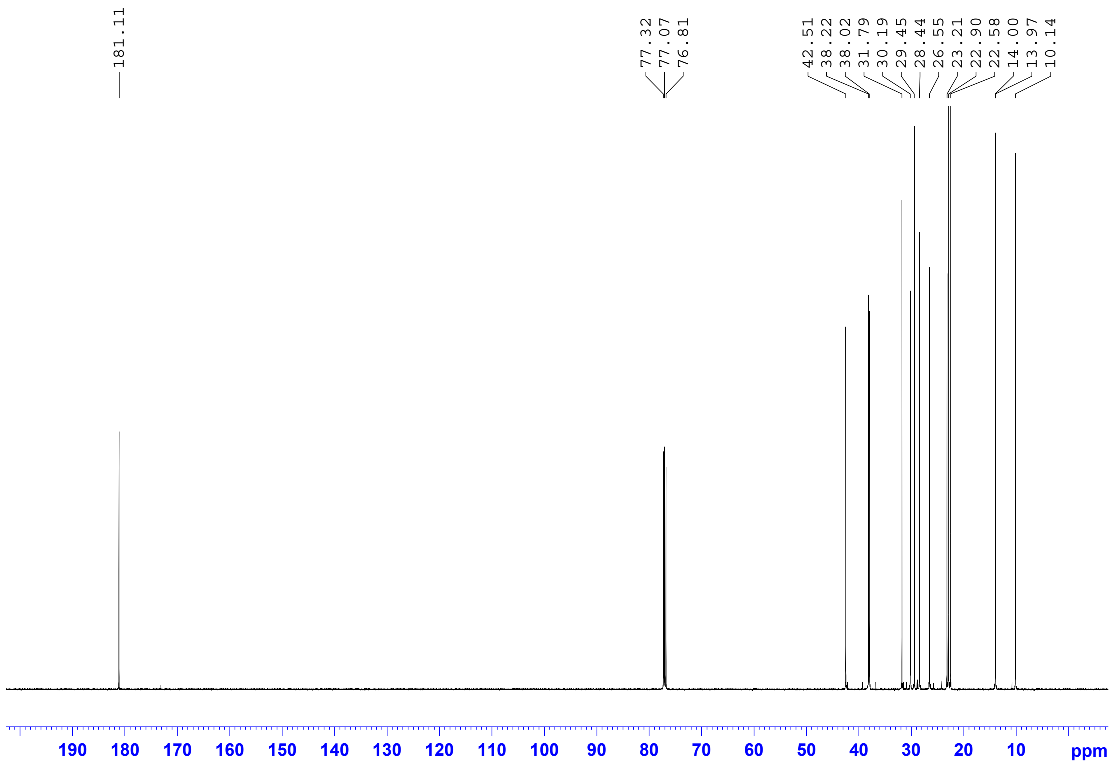

Figure S6: ^{13}C NMR analysis of [EHA][C7]

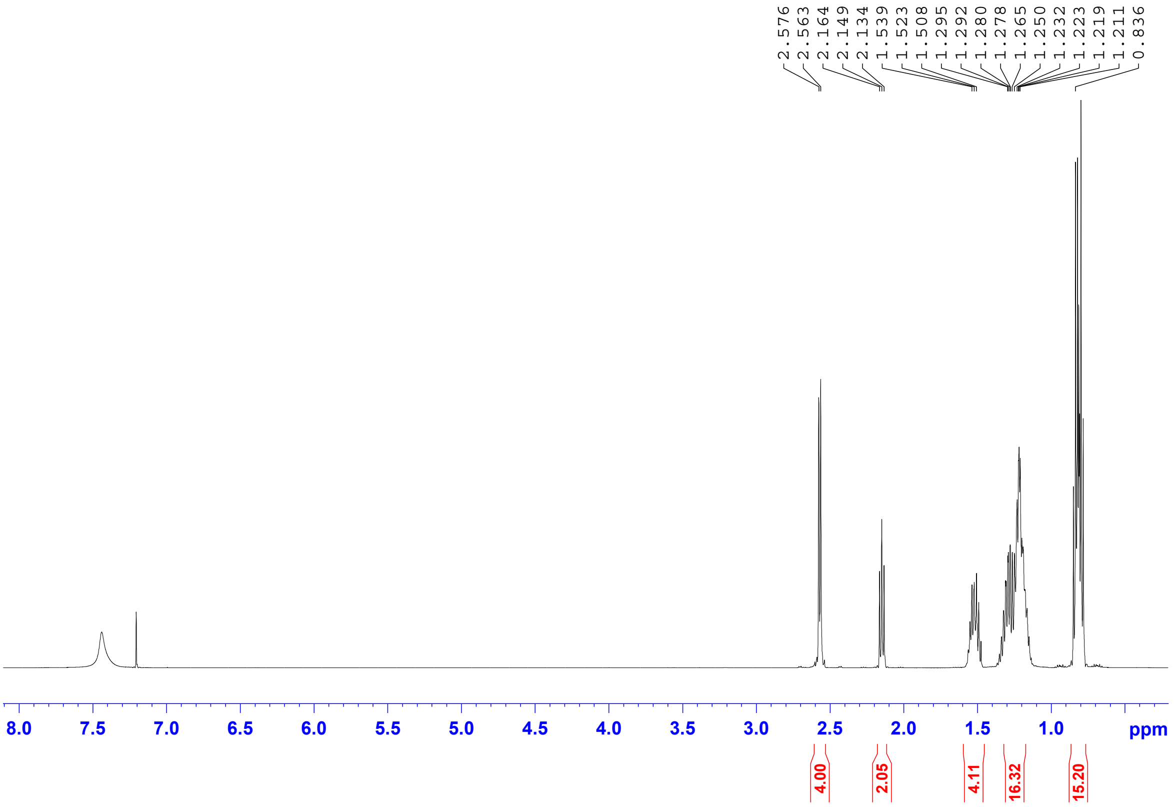

Figure S7: ^{1}H NMR analysis of [BEHA][C5]

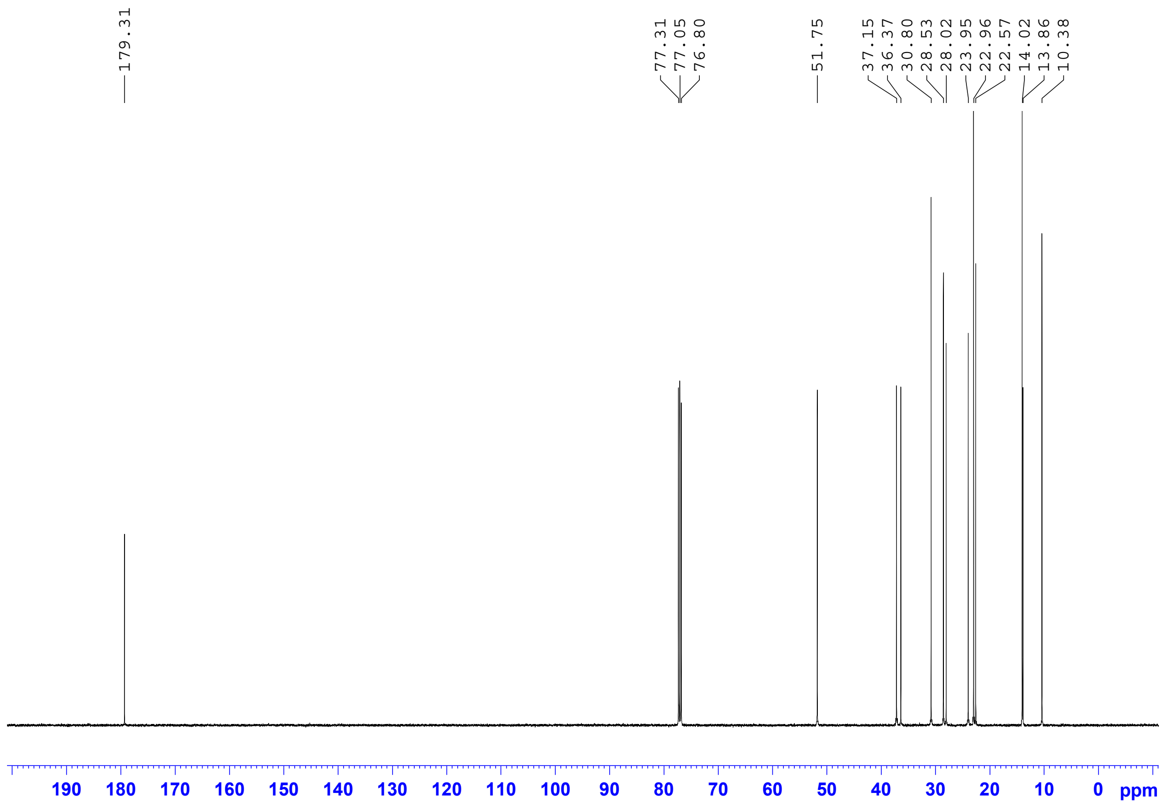

Figure S8: ^{13}C NMR analysis of [BEHA][C5]

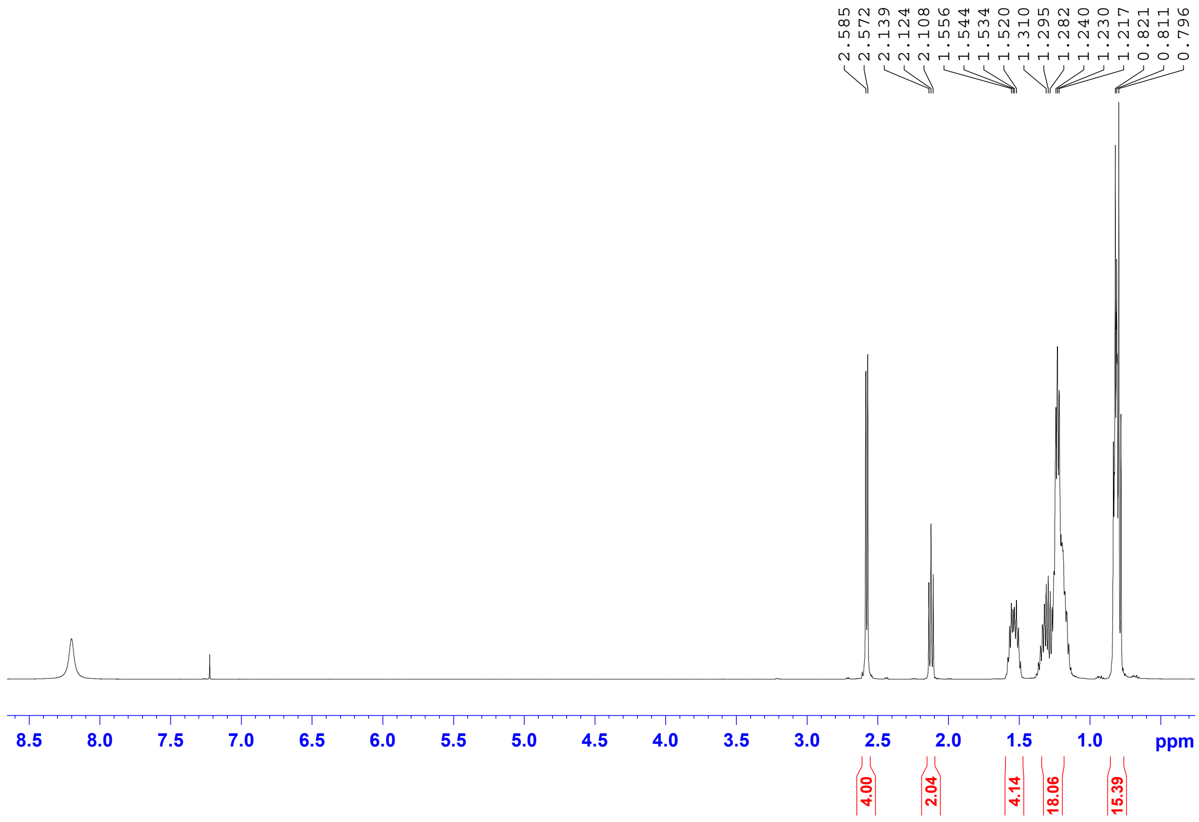

Figure S9: ^{1}H NMR analysis of [BEHA][C6]

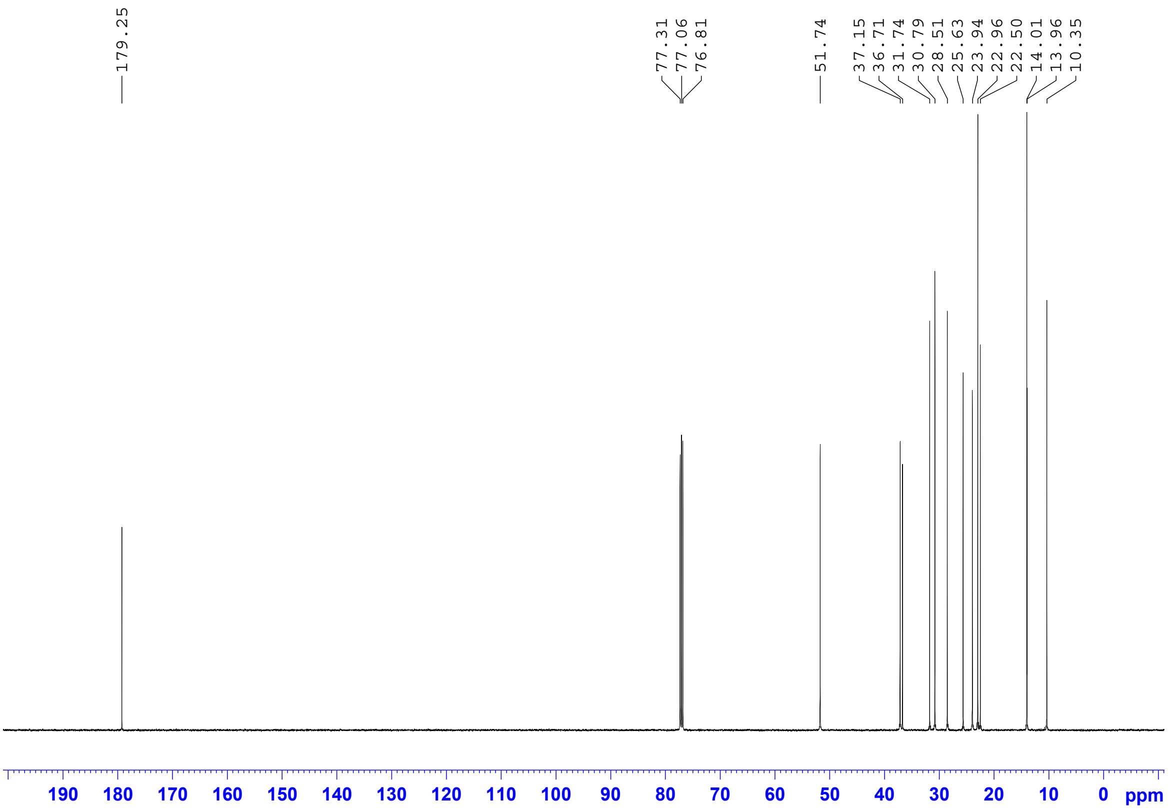

Figure S10: ^{13}C NMR analysis of [BEHA][C6]

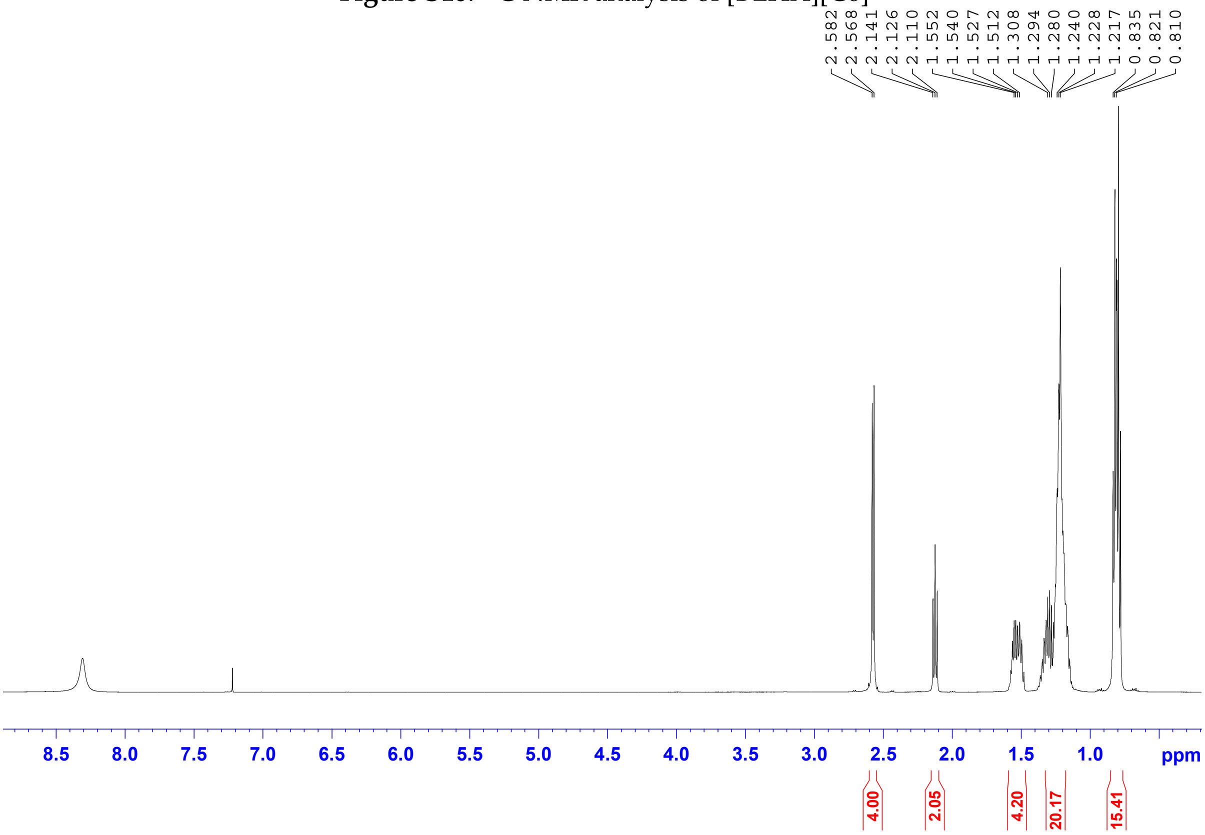

Figure S11: ^{1}H NMR analysis of [BEHA][C7]

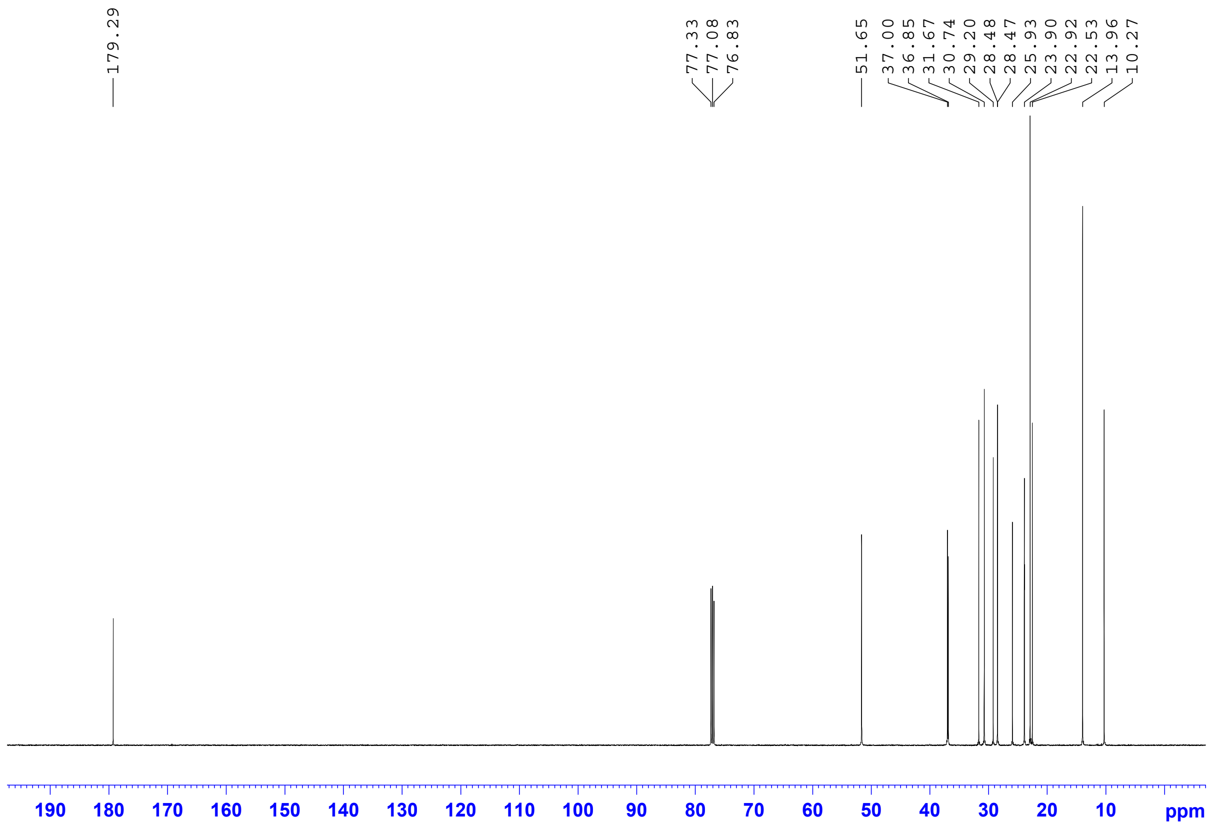

Figure S12: ^{13}C NMR analysis of [BEHA][C7]

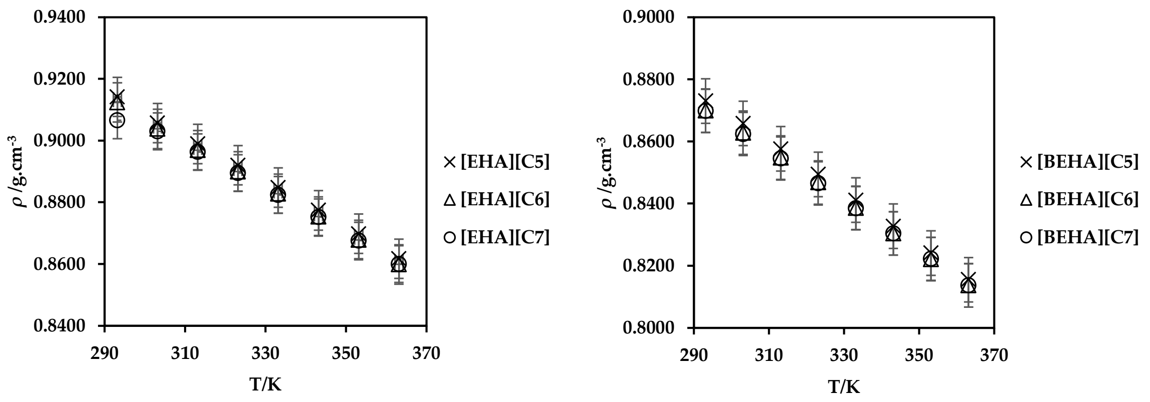

(a) **(b)**

Figure S13: Plots of density (ρ) values with standard errors of **(a)** [EHA][C5], [EHA][C6], [EHA][C7], and **(b)** [BEHA][C5], [BEHA][C6], [BEHA][C7] as a function of temperature.

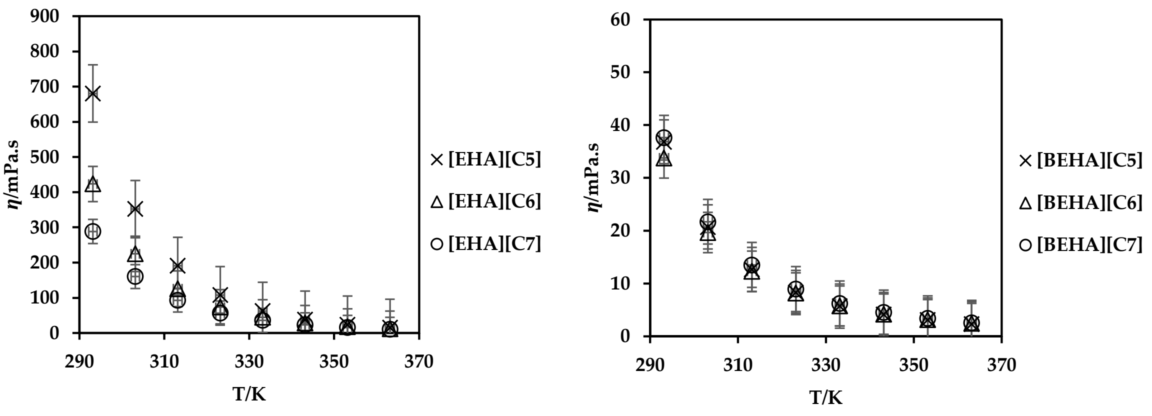

(a) **(b)**

Figure S14: Plots of viscosity (η) values with standard errors of **(a)** [EHA][C5], [EHA][C6], [EHA][C7], and **(b)** [BEHA][C5], [BEHA][C6], [BEHA][C7] as a function of temperature.

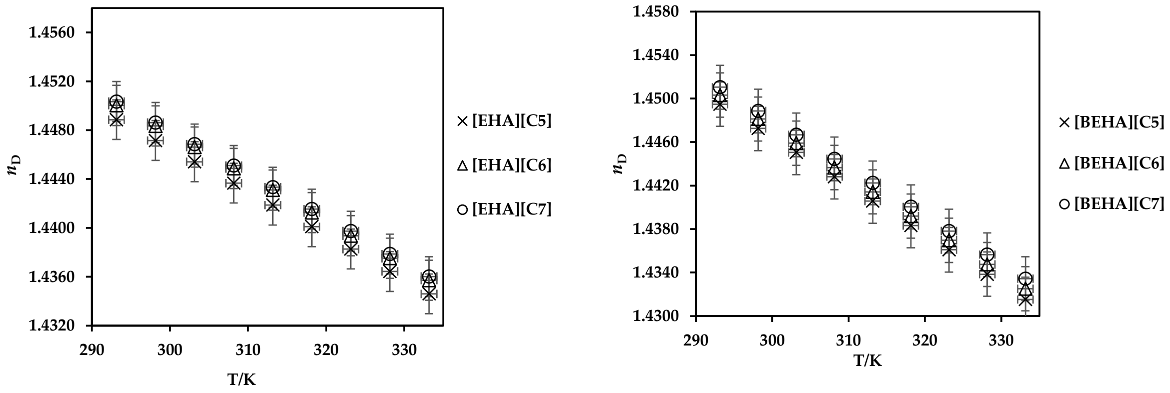

(a) **(b)**

Figure S15: Plots of refractive index (n_D) values with standard errors of **(a)** [EHA][C5], [EHA][C6], [EHA][C7], and **(b)** [BEHA][C5], [BEHA][C6], [BEHA][C7] as a function of temperature.
